# Impact assessment of increasing renewable energy penetration on voltage instability tendencies of power system buses using a QV-based index

**DOI:** 10.1038/s41598-023-36843-5

**Published:** 2023-06-16

**Authors:** Bukola Babatunde Adetokun, Christopher Maina Muriithi, Joseph Olorunfemi Ojo, Oghenewvogaga Oghorada

**Affiliations:** 1 Department of Electrical and Electronics Engineering, Nile University of Nigeria, Abuja, Nigeria; 2 Department of Electrical Engineering, Murang’a University of Technology, Murang’a, Kenya; 3 Department of Electrical and Computer Engineering, Tennessee Technological University, Cookeville, TN USA; 4 Present Address: Engineering Performance and Monitoring Division, Nigerian Electricity Regulatory Commission, Plot 1387, Cadastral Zone A00, Central Business District, Abuja, FCT Nigeria

**Keywords:** Electrical and electronic engineering, Renewable energy

## Abstract

This paper presents a QV-based approach called Critical Voltage-Reactive Power Ratio (CVQR) index to assess the voltage instability tendencies of power system buses with increase in renewable energy (RE) penetration within the power system. The buses are thus ranked according to the order in which they are impacted by increase in renewable energy penetration. Simulations were performed using DIgSILENT PowerFactory and result analyses were done with MATLAB. The developed CVQR index has been employed to assess the effect of increasing RE generation on grid voltage stability. This index provides information on the voltage instability tendencies of all non-slack buses of the RE-integrated grid and the buses are ranked from the weakest to the strongest. The rankings obtained from the developed CVQR has been compared with five commonly-used indices and the result of the comparison verifies the accuracy of the proposed index. IEEE 14-bus and IEEE 39-bus New England systems have been used to evaluate the proposed CVQR index and various scenarios of RE system combinations and placements have been considered. Voltage collapse condition is indicated whenever the CVQR index associated with any bus becomes positive (CVQR > 0). This index can as well be applied to other power system networks. The overall ranking of the buses based on the CVQR index can provide insights on the most appropriate location for large inductive loads or compensating devices, which can either absorb or inject reactive power into the power system, thereby influencing the system’s voltage stability.

## Introduction

The harnessing of clean and renewable energy (RE) resources for electricity generation has become a major research focus globally. There has been a significant rise in investments and the development of clean and renewable energy systems due to the need to preserve the environment from the effects of global warming and the need to meet the increasing energy demands^1,2^. Developing countries such as Kenya in Sub-Sahara Africa also utilise clean and renewable energy sources, which include geothermal, wind and hydro. These constitute the major percentage of the total installed generation capacity. It is therefore obvious that the subject of renewable energy integration to existing power grid has gained considerable attention globally^3–9^.

The extent to which clean and renewable energy integration can be achieved has been a subject of debate, particularly as it relates to whether 100% renewable energy penetration can be achieved or not^10,11^. Several researchers have discussed the possibilities and challenges of attaining a 100% renewable grid^11–13^ The authors in^11^ have presented some evidence against the arguments put forth in^10^, indicating that a 100% RE grid is both economically viable and technologically feasible. However, deliberate policies, clear-cut roadmaps and incentive regulations must be put in place in order to achieve this possibility^14,15^. Some have also shown that voltage stability and power quality concerns are the main factors that can limit the levels of renewable energy integration in power systems^16^.

Voltage stability is an important consideration for increased RE integration. Thus, this paper investigates scenarios of increased RE (wind and solar photovoltaic (PV)) integration and the resulting effect on the voltage stability of the power system. In this work, the Critical Voltage-Reactive Power Ratio (CVQR) index has been developed to assess the impact of increased RE integration on the voltage instability tendencies of power system buses.

Various investigations have been performed in order to study the effects of increased RE integration on power system stability using different voltage stability indices^17–29^. Voltage stability index is an important tool for power system planners, engineers, researchers and operators, which can provide insightful information on voltage stability monitoring, voltage instability prediction and prevention^30,31^.

## Related works and literature review

Several authors have developed line and bus voltage stability indices for power system using different approaches^32,33^. Table 1 provides comparisons of bus voltage stability indices. A review of different voltage stability indices has been carried out in^31,34–36^ and an exhaustive tabular comparison and classification of various indices has been presented in^31,36–38^. In particular, the authors of^31^, having recently carried out one of the most thorough review on voltage stability indices developed in the last thirty years, highlighted some inconsistencies among voltage stability indices due to contradictory results obtained for specific applications. This is because most of the developed indices are more generalized in application and may therefore be somewhat inaccurate for specific applications. As an illustration, some works have been carried out on the IEEE 39-bus system used as a case study in this work in order to determine the weakest load bus and to rank the buses accordingly. The authors in^39^ identified bus 15 as the critical bus of the IEEE 39-bus system based on *L* and *L*^***′***^ indices. In^40^, bus 12 was determined as the weakest bus using modal analysis (eigenvalue) method, reactive power margin index and a multi-criteria voltage stability index called ideal point method developed by the author. In^41^, the author used the ratio of standard deviation to mean of voltage magnitude to also identify bus 12 as the weakest bus in the IEEE 39-bus system and compared their result with Relative Voltage Vulnerability Index proposed in^42^ which also determined bus 12 as the weakest bus. However, there are slight variations in the overall bus rankings of power systems as indicated in^31^ and^41^.

**Table 1:** Features of some voltage stability indices.

S/N	Index name	Index type	Concept used for development	Defining equation	Instability/voltage collapse condition
1	Line stability index, L_mn_^43^	Line	Power flow in a two-bus power system	$L_{mn} = \frac{{4XQ_{r} }}{{(V_{s} \sin (\theta - \delta ))^{2} }}$	*L*_*mn*_ > 1
2	Fast voltage stability index (FVSI)^44^	Line	Power flow in a two-bus power system	$FVSI = \frac{{4Z^{2} Q_{r} }}{{V_{S}^{2} X}}$	*FVSI* > 1
3	Line stability factor (LQP)^45^	Line	Power flow in a two-bus power system	$$LQP = \frac{4X}{{V_{s}^{2} }}\left( {\frac{{XP_{s}^{2} }}{{V_{s}^{2} }} + Q_{r} } \right)$$$$LQP = \frac{4X}{{V_{s}^{2} }}\left( {\frac{{XP_{s}^{2} }}{{V_{s}^{2} }} + Q_{r} } \right)$$	LQP > 1
4	Voltage stability margin (VSM)^46^	Line	Maximum power transfer theroem	$$\begin{gathered} VSM = \frac{{S_{cr} - S_{L} }}{{S_{cr} }}; \hfill \\ S_{cr} = \frac{{V_{s}^{2} }}{{2Z\left[ {1 + \cos (\theta - \delta )} \right]}} \hfill \\ \end{gathered}$$	VSM < 0
5	Line Collapse Proximity Index (LCPI)^47^	Line	Voltage quadratic equation	$LCPI = \frac{{4\left| A \right|\cos \alpha \left( {P_{r} \left| B \right|\cos \beta + Q_{r} \left| B \right|\sin \beta } \right)}}{{(V_{s} \cos \alpha )^{2} }}$	LCPI > 1
6	Voltage Collapse Proximity Index (VCPI)^48^	Line	The voltage drop across the Thevinin impedance is equal to the load voltage at the point of voltage collapse	$VCPI = V_{r} \cos \delta - 0.5V_{s}$	VCPI < 0
7	Voltage stability index (VSI_bus_)^49^	Bus	Maximum power transfer theorem	$VSI_{bus} = \left[ {1 + \left( {\frac{{I_{i} }}{{V_{i} }}} \right)\left( {\frac{{\Delta V_{i} }}{{\Delta I_{i} }}} \right)} \right]^{\alpha }$	VSI_bus_ < 0
8	Equivalent node voltage collapse index (ENVCI)^50^	Bus	Equivalent local network model and equivalent system model	$ENVCI = 2\left( {e_{k} e_{n} + f_{k} f_{n} } \right) - \left( {e_{k}^{2} + f_{k}^{2} } \right)$	ENVCI < 0
9	Predicting the voltage collapse index^51^	Bus	Power flow solutions	V/V_o_	Extremely small value
10	L-index^52,53^	Bus	Power flow solutions	$L = \mathop {\max }\limits_{{j \in \alpha_{L} }} \left\{ {L_{j} } \right\} = \mathop {\max }\limits_{{j \in \alpha_{L} }} \left| {1 - \frac{{\sum\limits_{{i \in \alpha_{G} }} {F_{ji} V_{i} } }}{{V_{j} }}} \right|$	L > 1
11	Bus participation factor (BPF)^54^	Bus	Modal analysis	Details in the reference	Using a power system simulation tool
12	Voltage stability factor (VSF)^55,56^	Bus	Two-bus power flow	$VSF_{total} = \sum\limits_{m = 1}^{k - 1} {\left( {2V_{m + 1} - V_{m} } \right)}$	VSF = 0 at the collapse point
13	Voltage collapse prediction index (VCPI)^57^	Bus	Power flow solutions	$$VCPI_{j} = \left| {1 - \frac{{\sum\limits_{\begin{subarray}{l} m = 1 \\ m \ne j \end{subarray} }^{N} {V^{\prime}_{m} } }}{{V_{j} }}} \right|$$	VCPI_jth bus_ = 1

The indices developed in the aforementioned studies and in most studies concentrate on the ranking of only load buses. Whereas in some systems such as the IEEE 39-bus New England system, the weakest bus may not necessarily be a load bus. Thus, the bus ranking proposed in this study includes all types of buses apart from the slack bus. This provides a more comprehensive insight on voltage stability of the system. This work therefore presents a specific voltage instability tendency index called Critical Voltage-Reactive Power Ratio (CVQR) for power system with increased RE penetration.

Renewable energy penetration level (PL) has been defined as the ratio of the active power generated from all RE systems to the total active power generated by all sources. This is the same as the definition utilised in^29,58^ and the instantaneous PL in^59^. The reactive power capability of the synchronous generators and the renewable energy systems are put into consideration in this study. The reactive power characteristics of synchronous generators and solar photovoltaic system are already detailed in^60^.

The novelty and main contributions of this paper can be summarized as follows:

- This paper has explored the concept of QV curve analysis to develop a voltage instability tendency measure called CVQR.
- This index has been used to evaluate the impact of increased renewable energy PL on the voltage instability tendencies of power system buses as the RE penetration level increases.
- All non-slack buses can be ranked based on the values of the respective CVQR associated with them. The ranking provided with the use of CVQR index is not limited to only load buses as in other studies, but all non-slack buses are included. Thus, the CVQR-based ranking provides better insights into voltage stability of RE-integrated grid as it gives information on the voltage instability tendency of each power system bus.
- The performance of CVQR index has been compared with five commonly-used bus voltage stability indices and the results of the comparison verifies the accuracy of this developed index.

The rest of this paper is arranged as follows: Section “Description of renewable energy sources” presents a brief description of the RE sources considered in this work and their parameters. Section “Development of the proposed QV-based CVQR index” provides an overview of QV analysis, the derivation of QV equation for a simple two-bus system and the development of the CVQR index. Section “Application of the developed CVQR index” presents the applications of the CVQR index and the study is concluded in Section “Conclusion”.

## Description of renewable energy sources

Doubly-Fed Induction Generator (DFIG)-based wind energy conversion system (WECS) and PV systems have been considered in this work. Detailed modelling of DFIG-based WECS and solar PV systems are already provided in^29^. The penetration level of RE sources used in this work is taken as the ratio of the active power generated by RE sources to the active power generated by all sources.

The parameters of DFIG-based Wind Energy Conversion System and the large-scale solar PV system used in this work are presented in Tables 2 and 3 respectively.

**Table 2:** Parameters of 2 MW DFIG wind turbine generator in DIgSILENT PowerFactory.

Parameters	Value
Rated voltage	0.69 kV
Rated apparent power	2.222 MVA
Rated power factor	0.9
Nominal speed	1782.183 rpm
Number of pole pairs	2
Stator resistance	0.01 p.u
Stator reactance	0.1 p.u
Rotor resistance	0.01 p.u
Rotor reactance	0.1 p.u
Magnetising reactance	3.5 p.u
Moment of inertia	75 kg m^2^

**Table 3:** Large-scale solar PV plant parameters in DIgSILENT PowerFactory for one unit.

Parameters	Value
Rated voltage	0.69 kV
Rated apparent power	110 MVA
Rated power factor	1.0
Max. reactive power operational limit	0.43 p.u
Min. reactive power operational limit	− 0.43 p.u

## Development of the proposed QV-based CVQR index

This section presents the basics of QV curve, the derivation of QV equation for a simple two-bus system and the development of the proposed CVQR index.

### Overview of QV analysis

QV analysis is useful for voltage stability studies of power systems. The curve, which shows the variation of each bus voltage with respect to the reactive power injected at the same bus is called QV curve. The curve gives the reactive power required at a specified bus to maintain a certain voltage at the same bus. The QV curve provides the steady state voltage stability margins, such that the right side of the curve with positive slope signifies a stable operation, while the left side, with negative slope represents the unstable mode of the grid. This can also be explained from the fact that reactive power control devices are designed to satisfactorily operate when a rise in reactive power is accompanied by a corresponding rise in voltage^61^

**Figure 1:**
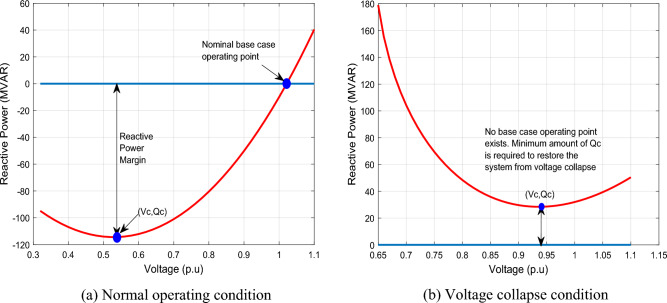
QV curve illustration for normal operating condition and voltage collapse condition.

Figure 1 illustrates the concept of QV curve. Figure 1a shows the QV curve for normal grid operation and Fig. 1b depicts the QV curve for voltage collapse condition. The critical operating point of voltage stability is indicated as (*V*_*C*_*, Q*_*C*_) in the figures. The minimum operating voltage is the critical voltage, *V*_*C*_, and the corresponding critical reactive power *Q*_*C*_, is the least quantity of reactive power needed to sustain the system to prevent voltage collapse. These values indicate the voltage stability limit of the grid.

### Derivation of QV equation for a simple two-bus power system

**Figure 2:**
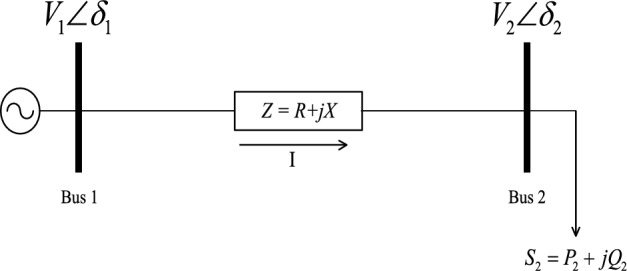
A simple two-bus system.

A basic equation that relates the bus voltage with the required reactive power is derived from the two-bus model of power system depicted in Fig. 2

The characteristic equation for this system can be expressed as:

1 $$I = \frac{{V_{1} \angle \delta_{1} - V_{2} \angle \delta_{2} }}{R + jX}$$

The complex power flow from bus 1 to bus 2 is given as:

2 $$P_{2} + jQ_{2} = V_{2} \angle \delta_{2} I^{ * } = \frac{{V_{1} V_{2} \angle \left( {\delta_{2} - \delta_{1} } \right) - V_{2}^{2} \angle 0}}{R - jX}$$

Let *θ* = *δ*_*2*_*−δ*_*1*_ and equating the real and the imaginary parts of both sides of Eq. (2), we have

3 $$\begin{gathered} \sin \theta = \frac{{RQ_{2} - XP_{2} }}{{V_{1} V_{2} }} \hfill \\ \cos \theta = \frac{{RP_{2} + XQ_{2} + V_{2}^{2} }}{{V_{1} V_{2} }} \hfill \\ \end{gathered}$$

Squaring both sides of Eq. (3) and combining the two equations, with $\sin^{2} \theta + \cos^{2} \theta = 1$, we have

4 $$Q_{2}^{2} + \left( {\frac{{2XV_{2}^{2} }}{{Z^{2} }}} \right)Q_{2} + \left[ {P_{2}^{2} + \frac{{\left( {V_{2}^{2} - V_{1}^{2} + 2RP_{2} } \right)V_{2}^{2} }}{{Z^{2} }}} \right] = 0$$

where $$Z^{2} = R^{2} + X^{2}$$.

For bus 2 QV curve, $P_{2} ,Z{\text{ and }}V_{1}$ are kept constant and the solution of Eq. (4) becomes

5 $$Q_{2} = - \frac{1}{{Z^{2} }}\left( {XV_{2}^{2} \pm \sqrt {\left( {Z^{2} V_{1}^{2} - 2RP_{2} Z^{2} - R^{2} V_{2}^{2} } \right)V_{2}^{2} - P_{2}^{2} Z^{4} } } \right)$$

This derivation of the QV curve equations presented for a simple 2-bus system illustrates the solution feasibility for any practical *n*-bus power system.

### Development of CVQR index

This section presents the development of the CVQR index for an *n*-bus system. For each PL, the CVQR is the ratio of critical voltage, *V*_*C*_ (in p.u) to critical reactive power, *Q*_*C*_ (in p.u) for each bus *j* = 1:*n*, and it can be expressed as:

6 $$CVQR(i,j) = \frac{{V_{C(i,j)} }}{{Q_{C(i,j)} }}$$

where $V_{C(i,j)}$ represents the critical voltage for bus *j* at the *i*th penetration level and $Q_{C(i,j)}$ represents the critical reactive power for bus *j* at the *i*th penetration level*.*

Since the critical reactive power is negative for normal operating conditions and positive when voltage collapse occurs, CVQR index associated with all the system buses are negative for normal operating conditions. The more negative the CVQR of a specified bus, the more unstable the bus becomes. Thus, when all the CVQR values of an *n*-bus power system are negative, the critical bus (CB) can be identified as:

7 $$CB_{CVQR} = \min \left\{ {\frac{{V_{c1} }}{{Q_{c1} }},\frac{{V_{c2} }}{{Q_{c2} }},\frac{{V_{c3} }}{{Q_{c3} }}, \ldots \frac{{V_{cn} }}{{Q_{cn} }}} \right\}$$

If the CVQR index associated with any bus is positive (CVQR > 0), then this indicates a voltage collapse condition of the grid.

**Figure 3:**
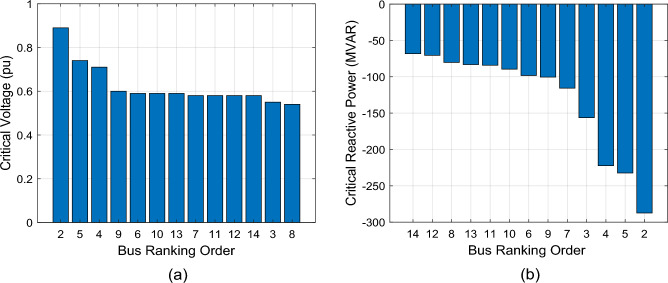
Critical voltage and reactive power margin of IEEE 14-bus system.

The CVQR provides a more comprehensive and accurate index to identify weak buses than utilizing only critical voltage level or only reactive power margin of buses. To illustrate this, we present the identification of weak buses based on critical voltage level, reactive power margin and the CVQR. Figure 3 shows the bar chart of the critical voltage and reactive power margin of each bus of the IEEE 14-bus test system. Ranking of the buses based on the critical voltage value is shown in Fig. 3a while the ranking based on the reactive power margin is shown in Fig. 3b. The higher the critical voltage, the higher the voltage instability tendency of the bus. However, buses with lower reactive power margin are considered weak because the reactive power margin measures the maximum reactive power load that can be accommodated at a bus above which the system will experience voltage collapse. It can be observed from Fig. 3a and b that the ranking based on critical voltage significantly differ from that of reactive power margin.

Since a higher critical voltage connotes higher tendency for voltage instability and a lower reactive power margin implies the same, this paper therefore proposes a more comprehensive index called Critical Voltage-Reactive Power Ratio (CVQR) index, which is the ratio of *V*_*C*_ (in p.u) to *Q*_*C*_ (in p.u). If any bus has a positive CVQR value, this indicates a condition of voltage collapse of the grid. A minimum amount of the critical reactive power of such bus(es) must be supplied at the respective bus(es) to return the system to normal operation. If the CVQR value is negative for all the buses, then the voltage instability tendency ranking of the system buses can be obtained. In this case, the more negative the CVQR value of a bus, the weaker the bus and the higher the voltage instability tendency at that bus. For instance, if the CVQR index of bus *j* is − 0.5 and that of bus *k* is − 1.5 then bus *j* is a stronger bus than bus *k*.

**Figure 4:**
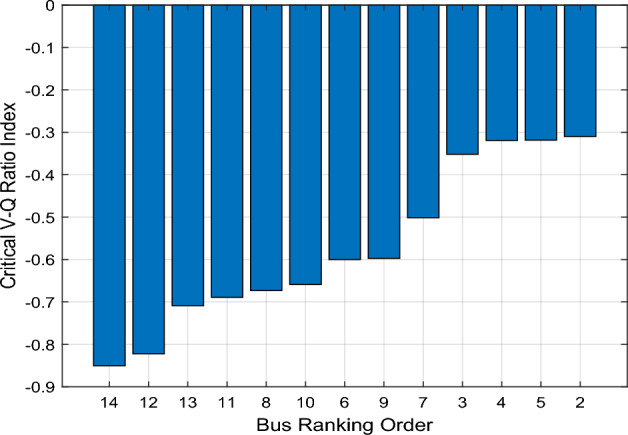
Critical V–Q ratio index ranking of IEEE 14-bus system.

Figure 4 shows the CVQR index ranking of the non-slack buses for the base case, when no renewable energy conversion system is integrated into the system. The ranking is from the weakest to the strongest bus. This shows that buses 14 and 12 are the weakest while buses 2 and 5 are the strongest buses.

In order to verify the accuracy of the proposed CVQR index, Table 4 shows the comparison of CVQR index ranking with respect to five commonly-used bus voltage stability indices, namely, L index^62^, Bus Participation Factor (BPF) associated with the minimum eigenvalue^63^, Reactive Power Margin (RPM) index, Voltage Stability Factor (VSF)^31^, and V/Vo index^31^. The load buses of the 14-bus test system are ranked using these indices. The table shows that the CVQR index ranking is the same with those obtained from *Lj*, BPF and RPM indices, whereas, there are variations in the ranking obtained from VSF and *Vj/Vo* indices. The inherent inaccuracies of VSF for power systems beyond 2-bus system upon which its derivation was based is already well-established in^31^. Thus, CVQR is a reliable index to evaluate the voltage instability tendency ranking of each power system bus.

**Table 4:** Ranking of IEEE 14-bus system load buses: comparison of CVQR index with commonly-used indices.

CVQR index		L-index		BPF index		RPM index		VSF index		V/Vo index	
Bus	CVQR	Bus	Lj	Bus	BPF	Bus	RPM	Bus	VSF	Bus	V/Vo
14	− 0.85056	14	0.222	14	0.122	14	68.19	12	1.085044	14	0.912321
12	− 0.82246	12	0.194	12	0.118	12	70.52	14	1.076698	10	0.925681
13	− 0.70905	13	0.138	13	0.114	13	83.21	9	1.067908	13	0.926024
11	− 0.68941	11	0.099	11	0.112	11	84.13	10	1.060878	9	0.929802
10	− 0.65907	10	0.079	10	0.108	10	89.52	13	1.060002	12	0.930355
9	− 0.59725	9	0.066	9	0.098	9	100.46	11	1.045101	11	0.931420
7	− 0.50147	7	0.049	7	0.078	7	115.66	5	1.016568	4	0.949080
4	− 0.31949	4	0.037	4	0.024	4	222.23	4	1.004157	7	0.949303
5	− 0.31838	5	0.031	5	0.020	5	232.43	7	0.976958	5	0.954419

## Application of the developed CVQR index

The performance of the CVQR index is tested using the IEEE 14-bus and 39-bus systems. This section therefore analyses the effects of increased renewable energy integration on the overall voltage instability tendency at each power system bus. This has been evaluated using the developed CVQR index. The buses are therefore ranked from the weakest to the strongest based on this analysis.

### Case study of the IEEE 14-bus system

**Figure 5:**
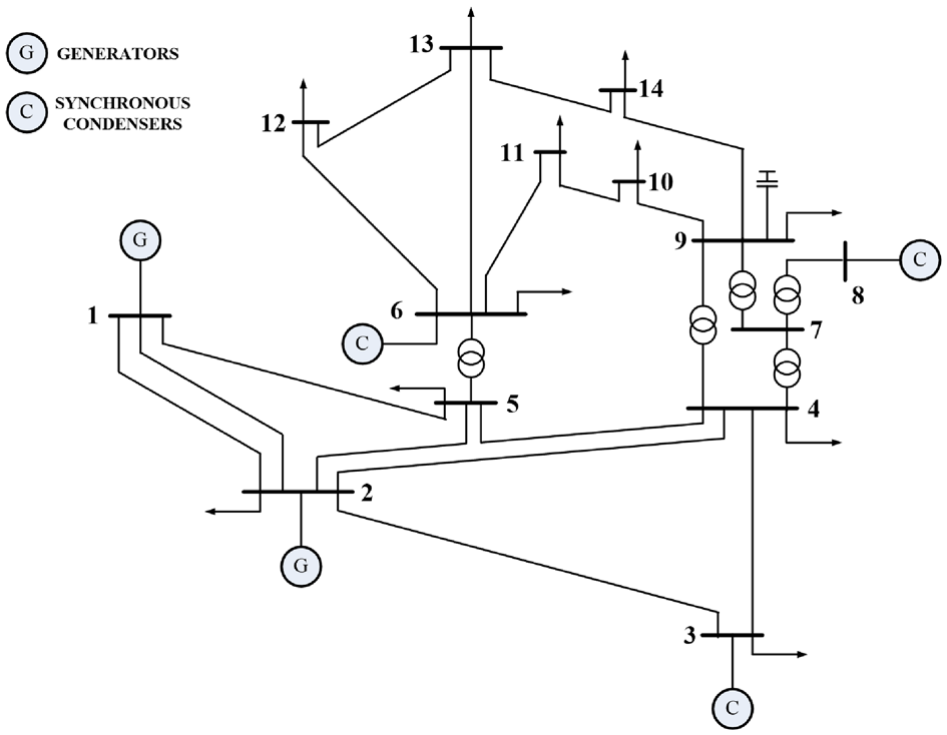
IEEE 14-bus network diagram.

Various scenarios of renewable energy mix and locations are studied using the IEEE 14-bus test system. The one-line diagram of IEEE 14-bus system is shown in Fig. 5 and its parameters are provided in^64^.

#### First scenario: DFIG-based WECS placed at bus 14

This section presents the influence of increased wind energy penetration on the voltage instability tendency of the power system as indicated by the CVQR index. The DFIG-WECS is connected to the system via bus 14. The PL is increased from 0.0 to 350 MW in steps of 50 MW. The 350 MW corresponds to 95.3% PL.

**Figure 6:**
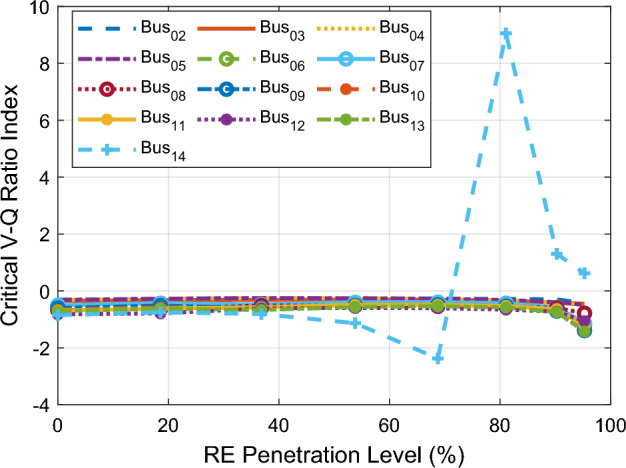
CVQR index for first scenario: DFIG-based WECS placed at bus 14.

Figure 6 shows the CVQR voltage instability tendency index of the system buses as the PL increases. This provides a comprehensive insight on the voltage stability status of the grid at each PL. It can be observed from the figure that the system experiences voltage collapse at 81.08% PL and above as indicated by the positive value of CVQR index for bus 14. If the CVQR value for any bus becomes positive at any PL, it indicates that there is occurrence of voltage collapse of the system at that PL. Table 5 shows the voltage instability tendency ranking of the system buses based on the cumulative CVQR index of each bus for all PL.

**Table 5:** CVQR-based ranking of non-slack buses for first scenario.

Rank	Bus #	Cumulative CVQR
1	14	5.0582
2	12	− 5.8724
3	13	− 5.8441
4	10	− 5.3961
5	9	− 5.3481
6	11	− 5.2561
7	8	− 4.8034
8	6	− 4.7925
9	7	− 4.3416
10	3	− 2.7935
11	4	− 2.6202
12	5	− 2.5822
13	2	− 2.4228

#### Second scenario: solar PV placed at bus 12 and DFIG-WECS at bus 14

In this scenario, the integration of PV system and DFIG-based WECS placed at bus 12 and 14 respectively is considered. The impact of increasing the penetration level of these RE systems is investigated in this section using CVQR index. In this scenario, the penetration level is increased from 0.0 MW PL to 303.2 MW, which corresponds to 99.97% PL for this case. This value is determined by load flow of the system. The RE mix considered in this case are:

- No RE generation ≡ 0% PL
- 50 MW DFIG-WECS + 0 MW PV system ≡18.56% PL
- 50 MW DFIG-WECS + 50 MW PV system ≡ 37.15% PL
- 100 MW DFIG-WECS + 50 MW PV system ≡ 55.09% PL
- 100 MW DFIG-WECS + 100 MW PV system ≡ 71.65% PL
- 150 MW DFIG-WECS + 100 MW PV system ≡ 86.69% PL
- 150 MW DFIG-WECS + 150 MW PV system ≡ 99.19% PL
- 153.23 MW DFIG-WECS + 150 MW PV system ≡ 99.97% PL

**Figure 7:**
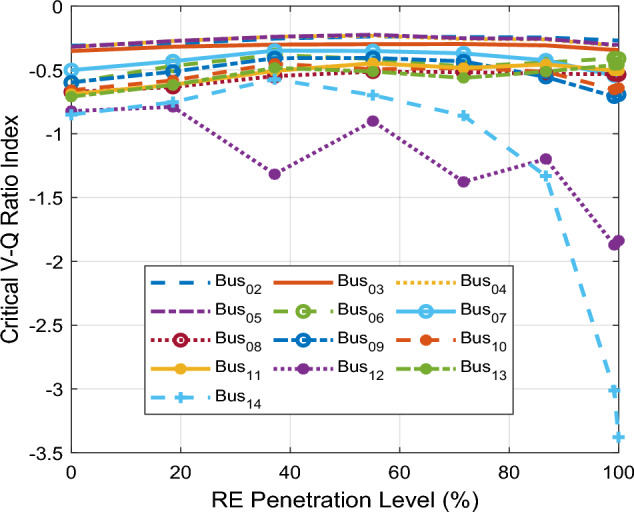
CVQR index for second scenario: PV system and DFIG-based WECS at bus 12 and bus 14 respectively.

Figure 7 shows the CVQR voltage instability tendency index of the system buses at the specified PL of the RE mix. This provides a comprehensive insight into the voltage stability status of the grid at each penetration level. The CVQR index of the buses at all specified PLs indicates that voltage collapse does not occur at any PL since all the CVQR values are negative. Moreover, the variation of the CVQR index with increasing PL shows that the CVQR index of all the buses (except bus 12) initially tends toward zero and then later becomes more negative as the PL approaches 100%. This implies that the increasing penetration of the RE systems at buses 12 and 14 initially enhances the voltage stability of the grid at lower PLs, but the grid tends towards voltage collapse as the PL increases and approaches 100%.

The voltage instability tendency ranking of each bus as indicated by their cumulative CVQR for all PLs is depicted in Table 6. The buses are ranked from the weakest to the strongest based on their cumulative CVQR. The ranking in Table 6 shows that bus 14 is still the weakest bus in the system followed by bus 12, and then buses 8 and 10. For this case, buses 2 and 5 remain the strongest buses in the system.

**Table 6:** CVQR-based ranking of non-slack buses for second scenario.

Rank	Bus #	Cumulative CVQR
1	14	− 11.452
2	12	− 10.113
3	8	− 4.486
4	10	− 4.479
5	13	− 4.319
6	9	− 4.315
7	11	− 4.211
8	6	− 3.600
9	7	− 3.508
10	3	− 2.561
11	4	− 2.203
12	5	− 2.169
13	2	− 2.122

#### Third scenario: DFIG-WECS placed at bus 2

This In this scenario, DFIG-WECS is placed at bus 2 considered as the strongest bus in the system. The penetration level is increased from 0.0 to 270.0 MW, which corresponds to 99.86% PL as obtained from the load flow of the system for this scenario.

**Figure 8:**
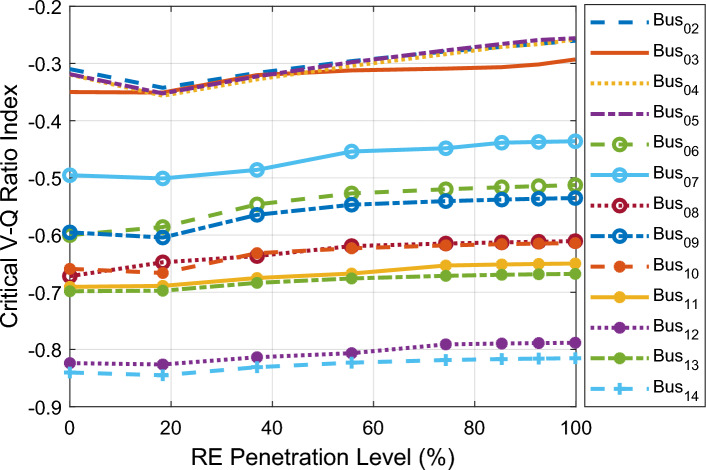
CVQR index for third scenario: DFIG-based WECS placed at bus 2.

Figure 8 depicts the CVQR index of the system buses with increment in DFIG-based WECS integration at bus 2. The CVQR index of the buses at all PLs indicates that voltage collapse does not occur at any PL since all the CVQR values are negative. The small negative CVQR values of the buses show that in this case, grid voltage stability is more enhanced than in the first two cases. As observed from the figure, the voltage stability of the grid is improved as the penetration level increases since the CVQR of the buses slightly tends towards zero as the PL increases. The CVQR-based ranking of the non-slack buses is shown in Table 7. The ranking indicates that on the overall, buses 14 and 12 are the weakest buses followed by buses 13 and 11. Buses 2 and 5 remain the strongest buses for this scenario also.

**Table 7:** CVQR-based ranking of non-slack buses for third scenario.

Rank	Bus #	Cumulative CVQR
1	14	− 6.606
2	12	− 6.428
3	13	− 5.432
4	11	− 5.327
5	10	− 5.043
6	8	− 5.025
7	9	− 4.462
8	6	− 4.323
9	7	− 3.696
10	3	− 2.543
11	4	− 2.387
12	5	− 2.349
13	2	− 2.341

#### Fourth scenario: solar PV placed at bus 5 and DFIG-WECS at bus 2

In this scenario, solar PV is placed at bus 5 and DFIG-WECS at bus 2. These are the strongest buses in the system. The penetration level is increased from 0.0 to 265.6 MW, which corresponds to 100% PL as obtained from the load flow of the system for this scenario. In this case, the following DFIG-WECS + PV system mix are utilised:

- 0 MW RE generation ≡ 0% PL
- 50 MW DFIG-WECS + 0 MW PV system ≡18.36% PL
- 50 MW DFIG-WECS + 50 MW PV system ≡ 37.23% PL
- 100 MW DFIG-WECS + 50 MW PV system ≡ 56.11% PL
- 100 MW DFIG-WECS + 100 MW PV system ≡ 75.31% PL
- 130 MW DFIG-WECS + 100 MW PV system ≡ 86.62% PL
- 150 MW DFIG-WECS + 100 MW PV system ≡ 94.10% PL
- 149.61 MW DFIG-WECS + 116 MW PV system ≡ 100.00% PL

**Figure 9:**
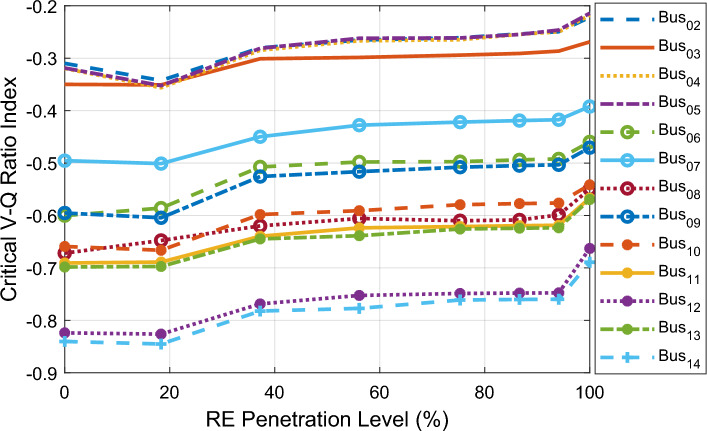
CVQR index for fourth scenario: solar PV placed at bus 5 and DFIG-based WECS at bus 2.

The CVQR index of the buses for this scenario is shown in Fig. 9. This index clearly indicates that voltage collapse does not occur at any PL since all the CVQR values are negative. In addition, the figure reveals that the voltage stability of the grid is most effectively improved as the PL increases since the CVQR of the buses significantly tends towards zero as the PL of the RE mix increases. Table 8 depicts the CVQR-based ranking of the non-slack buses for this case. The ranking also reveals that buses 14 and 12 are the weakest buses followed by buses 13 and 11. Buses 2 and 5 are the strongest buses in this scenario also.

**Table 8:** CVQR-based ranking of non-slack buses for fourth scenario.

Rank	Bus #	Cumulative CVQR
1	14	− 6.215
2	12	− 6.078
3	13	− 5.122
4	11	− 5.069
5	8	− 4.912
6	10	− 4.789
7	9	− 4.228
8	6	− 4.134
9	7	− 3.524
10	3	− 2.441
11	4	− 2.215
12	5	− 2.191
13	2	− 2.184

The analyses of from the four scenarios show that when the RE systems (DFIG-WECS and solar PV) are connected to the system via weak buses, voltage stability of the grid is initially enhanced at lower penetration levels but the system begins to tend towards voltage instability as the penetration level significantly increases and approaches 100%. However, when they are connected to the grid via the strongest buses, voltage stability is effectively enhanced as the penetration level increases.

### Case study of the IEEE 39-bus system

**Figure 10:**
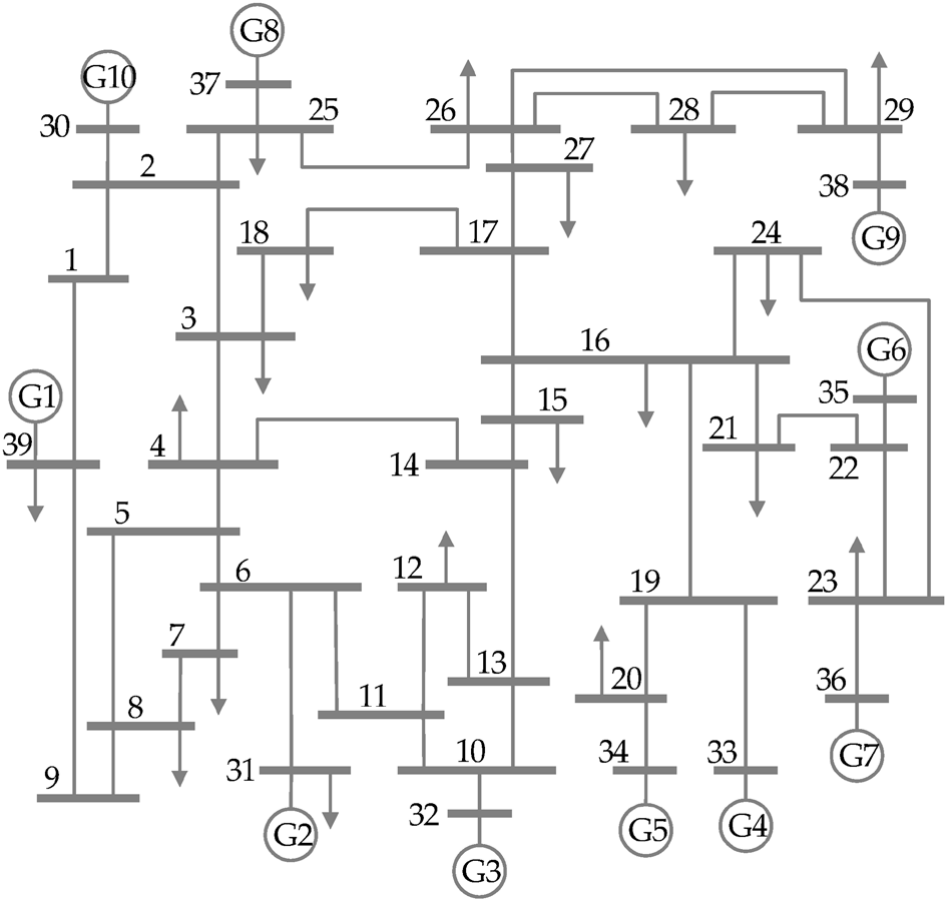
One-line diagram of IEEE 39-bus system^66^.

The CVQR index evaluation has been carried out in this section for the IEEE 39-bus system. As depicted in the one-line diagram of Fig. 10, this system consists of ten synchronous generators, nineteen loads, twelve tie-lines, twelve transformers and thirty-four transmission lines. The parameters of the test system are detailed in^65^. The tie-line buses are buses 1, 2, 5, 6, 9, 10, 11, 13, 14, 17, 19, and 22. Neither load nor generator is connected to these buses.

In establishing the ranking of non-slack buses of the 1EEE-39 bus system for the base case scenario when there is no RE integration, the proposed CVQR index in comparison with the reactive power margin (RPM) index for each bus is depicted in Table 9. The table shows that load bus 12 is the weakest load bus, which is in agreement with^40–42^. However, this work further reveals that that none of the load buses is the most critical bus in the network. Rather, as indicated in the table, the most critical bus in the system is bus 38, which is a generator-connected (PV) bus. The next six critical buses are also generator-connected buses. The equivalent RPM shows that the maximum reactive power that must not be absorbed from bus 38 for the system not to experience voltage collapse is 178.54 MVAR. For load bus 12, the RPM is 644.9 MVAR, which is less critical than the first seven highest-ranked buses. The rankings from CVQR and RPM are similar except for some slight variations because CVQR takes into account the magnitude of the critical voltage for each bus.

**Table 9:** Ranking of IEEE 39-Bus system non-slack buses: comparison of CVQR index with reactive power margin (RPM) Index.

Rank	CVQR index		RPM index		Rank	CVQR Index		RPM index	
	Bus	CVQR	Bus	RPM		Bus	CVQR	Bus	RPM
1	38	− 0.40887	38	178.54	20	6	− 0.05817	6	1168.99
2	36	− 0.13241	36	490.9	21	13	− 0.0576	22	1179.16
3	34	− 0.11269	34	532.43	22	5	− 0.05666	5	1182.43
4	39	− 0.10672	39	534.11	23	19	− 0.05568	19	1239.23
5	32	− 0.10203	32	568.47	24	21	− 0.05201	21	1288.28
6	37	− 0.101	37	594.07	25	14	− 0.04768	14	1321.34
7	33	− 0.09259	33	626.41	26	4	− 0.04734	4	1330.68
8	12	− 0.09149	12	644.9	27	26	− 0.0467	26	1413.27
9	29	− 0.09074	35	706.06	28	24	− 0.04558	27	1435.63
10	28	− 0.08797	28	807.12	29	15	− 0.04209	9	1481.26
11	35	− 0.08781	29	837.58	30	16	− 0.04069	15	1544.16
12	20	− 0.07056	20	892.92	31	27	− 0.0397	24	1557.56
13	23	− 0.06183	30	919.08	32	25	− 0.03784	1	1611.38
14	7	− 0.06098	7	1049.56	33	9	− 0.03646	25	1665.02
15	11	− 0.05969	8	1086.51	34	3	− 0.03524	18	1756.56
16	10	− 0.05925	23	1115.95	35	17	− 0.03488	16	1818.6
17	8	− 0.0589	11	1122.39	36	18	− 0.03416	3	1844.41
18	30	− 0.05875	10	1130.85	37	1	− 0.03289	17	1949.53
19	22	− 0.05852	13	1145.74	38	2	− 0.03066	2	1989.66

In the case considered for the IEEE 39-bus system with increasing RE penetration, the synchronous generators are successively replaced by DFIG-based WECS and solar PV. Thus, the locations of the RE generations are largely on the weak buses since the first seven weakest buses as identified by both the CVQR and the RPM indices are those to which the conventional generators are connected. The following DFIG-WECS + PV system mix are considered:

- 0 MW RE generation ≡ 0% PL
- 508 MW DFIG-WECS + 0 MW PV system ≡ 8.27% PL
- 508 MW DFIG-WECS + 540 MW PV system ≡ 17.06% PL
- 1158 MW DFIG-WECS + 540 MW PV system ≡ 27.64% PL
- 1158 MW DFIG-WECS + 1190 MW PV system ≡ 38.22% PL
- 2158 MW DFIG-WECS + 1190 MW PV system ≡ 54.48% PL
- 2158 MW DFIG-WECS + 2020 MW PV system ≡ 67.98% PL
- 2158 MW DFIG-WECS + 2270 MW PV system ≡ 72.05% PL
- 2718 MW DFIG-WECS + 2270 MW PV system ≡ 81.15% PL
- 2718 MW DFIG-WECS + 2797.63 MW PV system ≡ 89.72% PL
- 3350 MW DFIG-WECS + 2798.4 MW PV system ≡ 100.00% PL

**Figure 11:**
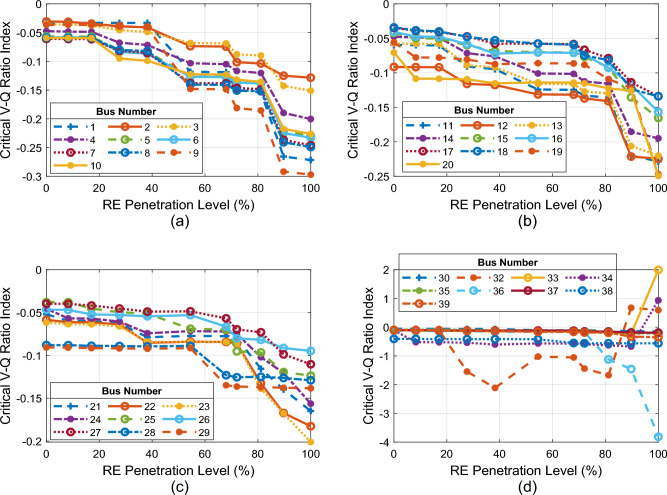
CVQR index for IEEE 39-bus system.

The CVQR index of each bus with increase in PL of DFIG-WECS + PV system is depicted in Fig. 11a–d. Figure 11a shows the CVQR for buses 1–10; Fig. 11b shows the CVQR for buses 11–20; Fig. 11c depicts the CVQR for buses 21–29 and Fig. 11d shows the CVQR for buses 30–39 except bus 31, which is the slack bus. The figures show that CVQR index of all the buses becomes more negative with increasing RE PL. This indicates that the voltage instability tendency of the system increase as the RE PL increases. However, the CVQR index for bus 32 exhibits an anomalous behaviour: it tends towards voltage instability at first then it becomes voltage stable as the PL increases.

With further increase in PL, the CVQR for bus 32 tends toward voltage instability again and ultimately, at 89.72% PL, it changes sign (becomes positive); thereby showing an occurrence of voltage collapse of the system.

The overall CVQR-based voltage instability tendency ranking of the non-slack buses of the IEEE 39-Bus New England System is depicted in Table 10. The ranking shows that the first seven weakest buses are generator-connected buses. The weakest load bus is bus 12. It can be observed that the most critical load buses are actually the buses closest to the critical generator buses as shown in the network diagram of Fig. 10. For instance, bus 12 is the weakest load bus and it is the closest to the most critical generator bus 32. In addition, bus 20 is the second weakest load bus and it is as well the closest load bus to the second most critical generator bus 34. Furthermore, the weakest tie-line buses 10, 11, 6, 5, 9, and 13 are linked to the most critical bus 32. In order to enhance the voltage stability of this RE-integrated grid and to prevent voltage collapse of the grid at higher PL, reactive power compensation such as those provided by shunt FACTS devices are required. They should be located at the most critical buses such as 32 and 34 or their nearest load buses or tie-line buses.

**Table 10:** CVQR-based voltage instability tendency ranking of the non-slack buses of the IEEE 39-bus New England system.

Rank	Bus no.	Cumulative CVQR	Rank	Bus no.	Cumulative CVQR	Rank	Bus no.	Cumulative CVQR
1	32	− 9.15304	14	28	− 0.90585	27	1	− 0.70618
2	34	− 4.66647	15	10	− 0.88554	28	21	− 0.66348
3	38	− 4.16367	16	11	− 0.88288	29	24	− 0.61495
4	36	− 2.30445	17	6	− 0.87536	30	15	− 0.57991
5	39	− 1.28082	18	5	− 0.86079	31	16	− 0.57392
6	33	− 1.2798	19	9	− 0.85262	32	25	− 0.55296
7	37	− 1.10044	20	13	− 0.84401	33	26	− 0.53442
8	12	− 1.04994	21	30	− 0.75098	34	2	− 0.52954
9	35	− 0.98648	22	19	− 0.74907	35	3	− 0.5183
10	20	− 0.97809	23	23	− 0.73323	36	18	− 0.48635
11	29	− 0.95706	24	4	− 0.7288	37	17	− 0.48106
12	8	− 0.92361	25	14	− 0.72375	38	27	− 0.46367
13	7	− 0.9217	26	22	− 0.7176			

## Conclusion

A Q–V based method to assess the voltage instability tendencies of each bus of RE-integrated grid has been presented in this study. A summary of this work is as follows:

- The Critical Voltage-Reactive Power Ratio (CVQR) was developed and utilised to assess the effect of increased RE generation on grid voltage stability. The CVQR provides information regarding the voltage instability tendencies of non-slack buses of the RE-integrated grid and the buses are thus ranked from the weakest to the strongest. The rankings obtained from the proposed CVQR has been compared with other commonly-used indices to verify its accuracy.
- The developed CVQR index has been evaluated on IEEE 14-bus and IEEE 39-bus systems. The four scenarios investigated for the 14-bus system show that when the RE systems (DFIG-WECS and solar PV) are connected to the system via weak buses, voltage stability of the grid is initially enhanced at lower penetration levels but the system begins to tend towards voltage instability as the penetration level significantly increases and approaches 100%. However, when they are connected to the grid via the strongest buses, voltage stability initially declines at lower PLs but begins to improve and thus effectively enhanced as the penetration level increases and approaches 100%.
- All non-slack buses are analysed and ranked using CVQR in this study. As seen in the IEEE 39-bus case study, the weakest bus 32 is a generator-connected bus and the weakest load bus (bus 12) is located closest to it. The weakest tie-line buses are also those connected to the weakest buses 32 and 34. Thus, the CVQR-based ranking presented in this study provides better insights into voltage stability of RE-integrated grid.
- The voltage instability tendency rankings of buses obtained from CVQR provides insight on the most suitable placement of high-impact reactive loads and FACTS devices, which can either absorb or inject reactive power into the power system as the renewable energy penetration level increases. In particular, in order to enhance the voltage stability of power grid and to prevent voltage collapse of the grid at higher RE penetration levels, reactive power compensation such as those provided by shunt FACTS devices are required. They should be located at the critical buses as determined by the CVQR index ranking.
- The issue of frequency deviation and generation-demand imbalance during renewable energy integration can be examined in a future study. Various fault scenarios can also be studied.

## Data Availability

All data generated or analysed during this study are included in this published article.

## References

[1] Adetokun BB, Muriithi CM, Ojo JO (2020). Voltage stability assessment and enhancement of power grid with increasing wind energy penetration. Int. J. Electr. Power Energy Syst..

[2] Nagamani C, Saravana-Ilango G, Reddy MJB, Rani MAA, Lakaparampil ZV (2015). Renewable power generation indian scenario: A review. Electr. Power Compon. Syst..

[3] Ayodele TR, Ogunjuyigbe ASO, Adetokun BB (2017). Optimal capacitance selection for a wind-driven self-excited reluctance generator under varying wind speed and load conditions. Appl. Energy.

[4] Ogunjuyigbe ASO, Ayodele TR, Adetokun BB (2017). Steady state analysis of wind-driven self-excited reluctance generator for isolated applications. Renew. Energy.

[5] Adetokun, B. B., Muriithi, C. M. & Ojo, J. O. Voltage stability analysis and improvement of power system with increased SCIG-based wind system integration. In*2020 IEEE PES/IAS PowerAfrica* 1–5 (2020).

[6] Blaabjerg F, Ionel DM (2015). Renewable energy devices and systems—state-of-the-art technology, research and development, challenges and future trends. Electr. Power Compon. Syst..

[7] Kerekes T, Séra D, Máthé L (2015). Three-phase photovoltaic systems: Structures, topologies, and control. Electr. Power Compon. Syst..

[8] Adetokun BB, Muriithi CM (2021). Impact of integrating large-scale DFIG-based wind energy conversion system on the voltage stability of weak national grids: A case study of the Nigerian power grid. Energy Rep..

[9] Adetokun BB, Ojo JO, Muriithi CM (2021). Application of large-scale grid-connected solar photovoltaic system for voltage stability improvement of weak national grids. Sci. Rep..

[10] Heard BP, Brook BW, Wigley TML, Bradshaw CJA (2017). Burden of proof: A comprehensive review of the feasibility of 100% renewable-electricity systems. Renew. Sustain. Energy Rev..

[11] Brown TW, Bischof-Niemz T, Blok K, Breyer C, Lund H, Mathiesen BV (2018). Response to ‘Burden of proof: A comprehensive review of the feasibility of 100% renewable-electricity systems’. Renew. Sustain. Energy Rev..

[12] Child M, Kemfert C, Bogdanov D, Breyer C (2019). Flexible electricity generation, grid exchange and storage for the transition to a 100% renewable energy system in Europe. Renew. Energy.

[13] Bogdanov D, Breyer C (2016). North-East Asian Super Grid for 100% renewable energy supply: Optimal mix of energy technologies for electricity, gas and heat supply options. Energy Convers. Manage..

[14] Gerbaulet C, von Hirschhausen C, Kemfert C, Lorenz C, Oei PY (2019). European electricity sector decarbonization under different levels of foresight. Renew. Energy.

[15] Matschoss P, Bayer B, Thomas H, Marian A (2019). The German incentive regulation and its practical impact on the grid integration of renewable energy systems. Renew. Energy.

[16] Tang Z, Hill DJ, Liu T (2017). Two-stage voltage control of subtransmission networks with high penetration of wind power. Control Eng. Pract..

[17] Mitra A, Chatterjee D (2013). A sensitivity based approach to assess the impacts of integration of variable speed wind farms on the transient stability of power systems. Renew. Energy.

[18] Noghreian E, Koofigar HR (2020). Power control of hybrid energy systems with renewable sources (wind-photovoltaic) using switched systems strategy. Sustain. Energy Grids Netw..

[19] da Costa JN, Passos-Filho JA, Mota-Henriques R (2019). Loading margin sensitivity analysis in systems with significant wind power generation penetration. Electr. Power Syst. Res..

[20] Chen L, Min Y, Dai Y, Wang M (2017). Stability mechanism and emergency control of power system with wind power integration. IET Renew. Power Gener..

[21] Krismanto AU, Mithulananthan N, Krause O (2018). Stability of renewable energy based microgrid in autonomous operation. Sustain. Energy Grids Netw..

[22] Refaat SS, Abu-Rub H, Sanfilippo AP, Mohamed A (2018). Impact of grid-tied large-scale photovoltaic system on dynamic voltage stability of electric power grids. IET Renew. Power Gener..

[23] Hung DQ, Mithulananthan N, Bansal RC (2014). Integration of PV and BES units in commercial distribution systems considering energy loss and voltage stability. Appl. Energy.

[24] Worighi I, Maach A, Hafid A, Hegazy O, Van-Mierlo J (2019). Integrating renewable energy in smart grid system: Architecture, virtualization and analysis. Sustain. Energy Grids Netw..

[25] Yaghoobi J, Islam M, Mithulananthan N (2018). Analytical approach to assess the loadability of unbalanced distribution grid with rooftop PV units. Appl. Energy.

[26] Dkhili N, Eynard J, Thil S, Grieu S (2020). A survey of modelling and smart management tools for power grids with prolific distributed generation. Sustain. Energy Grids Netw..

[27] Wu J-H, Wang H-Y, Wang W-Q, Zhang Q (2019). A Comprehensive evaluation approach for static voltage stability analysis in electric power grids. Electr. Power Compon. Syst..

[28] Ma J, Qiu Y, Kang S, Thorp JS (2017). Time-delay stability analysis of stochastic power system with wind power connection. Electr. Power Compon. Syst..

[29] Adetokun BB, Ojo JO, Muriithi CM (2020). Reactive power-voltage-based voltage instability sensitivity indices for power grid with increasing renewable energy penetration. IEEE Access.

[30] Furukakoi M, Adewuyi OB, Shah-Danish MS, Howlader AM, Senjyu T, Funabashi T (2018). Critical Boundary Index (CBI) based on active and reactive power deviations. Int. J. Electr. Power Energy Syst..

[31] Danish MSS, Senjyu T, Danish SMS, Sabory NR, Krishnan N, Mandal P (2019). A recap of voltage stability indices in the past three decades. Energies.

[32] Li S (2016). Sensitivity model of L index for steady-state voltage stability of wind power systems with doubly fed induction generators. Electr. Power Compon. Syst..

[33] Halilčević SS, Softić I (2016). The line and node voltage stability index presented through the porosity of high-voltage transmission lines. Electr. Power Compon. Syst..

[34] Prasad, A., Manmohan, A., Karthikeyan, S. P. & Kothari, D. P. Assessment on various node voltage stability indices—a review. In*2017 International Conference On Smart Technologies For Smart Nation (SmartTechCon)* 395–400 (2017).

[35] Oukennou, A. & Sandali, A. Assessment and analysis of Voltage Stability Indices in electrical network using PSAT Software. In *2016 Eighteenth International Middle East Power Systems Conference (MEPCON)* 705–710 (2016).

[36] Modarresi J, Gholipour E, Khodabakhshian A (2016). A comprehensive review of the voltage stability indices. Renew. Sustain. Energy Rev..

[37] Nageswa-Rao AR, Vijaya P, Kowsalya M (2021). Voltage stability indices for stability assessment: A review. Int. J. Ambient Energy.

[38] Salama HS, Vokony I (2022). Voltage stability indices–a comparison and a review. Comput. Electr. Eng..

[39] Chen H, Jiang T, Yuan H, Jia H, Bai L, Li F (2017). Wide-area measurement-based voltage stability sensitivity and its application in voltage control. Int. J. Electr. Power Energy Syst..

[40] Gao, P., Shi, L., Yao, L., Ni, Y. & Bazargan, M. Multi-criteria integrated voltage stability index for weak buses identification. In*2009 Transmission & Distribution Conference & Exposition: Asia and Pacific* 1–5 (2009).

[41] Yusuff, A. A. Voltage stability index based on standard deviation-mean ratio for identification of weak nodes. In*2017 IEEE AFRICON* 1255–1259 (2017).

[42] Lin, Y. Z., Shi, L. B., Ni, Y. X., Yao, L. Z. & Bazargan, M. Multi-criteria voltage vulnerability index based on data envelopment analysis. In *9th IET International Conference on Advances in Power System Control, Operation and Management (APSCOM 2012)* 1–5 (2012).

[43] Moghavvemi M, Omar FM (1998). Technique for contingency monitoring and voltage collapse prediction. IEE Proc. Gener. Trans. Distrib..

[44] Musirin, I. & Rahman, T. K. A. Novel fast voltage stability index (FVSI) for voltage stability analysis in power transmission system. In *Student Conference on Research and Development* 265–268 (2002).

[45] Singh, P., Parida, S. K., Chauhan, B. & Choudhary, N. Online voltage stability assessment using artificial neural network considering voltage stability indices. In *2020 21st National Power Systems Conference (NPSC)* 1–5 (2020).

[46] Deng, G., Sun, Y. & Xu, J. A new index of voltage stability considering distribution network. In*2009 Asia-Pacific Power and Energy Engineering Conference* 1–4 (2009).

[47] Tiwari R, Niazi KR, Gupta V (2012). Line collapse proximity index for prediction of voltage collapse in power systems. Int. J. Electr. Power Energy Syst..

[48] Liang, W., Yutian, L. & Zhaowen, L. Power transmission paths based voltage stability assessment. In*2005 IEEE/PES Transmission & Distribution Conference & Exposition: Asia and Pacific* 1–5 (2005).

[49] Haque, M. H. Use of local information to determine the distance to voltage collapse. In *2007 International Power Engineering Conference (IPEC 2007)* 407–412 (2007).

[50] Wang Y, Li W, Lu J (2009). A new node voltage stability index based on local voltage phasors. Electr. Power Syst. Res..

[51] Danish MSS (2015). Voltage Stability in Electric Power System: A Practical Introduction.

[52] Kessel P, Glavitsch H (1986). Estimating the voltage stability of a power system. IEEE Trans. Power Deliv..

[53] Hongjie J, Xiaodan Y, Yixin Y (2005). An improved voltage stability index and its application. Int. J. Electr. Power Energy Syst..

[54] Song Y, Hill DJ, Liu T (2019). State-in-mode analysis of the power flow Jacobian for static voltage stability. Int. J. Electr. Power Energy Syst..

[55] Sultana U, Khairuddin AB, Aman MM, Mokhtar AS, Zareen N (2016). A review of optimum DG placement based on minimization of power losses and voltage stability enhancement of distribution system. Renew. Sustain. Energy Rev..

[56] Kayal P, Chanda CK (2013). Placement of wind and solar based DGs in distribution system for power loss minimization and voltage stability improvement. Int. J. Electr. Power Energy Syst..

[57] Balamourougan V, Sidhu TS, Sachdev MS (2004). Technique for online prediction of voltage collapse. IEE Proc. Gener. Transm. Distrib..

[58] Eftekharnejad S, Vittal V, Heydt GT, Keel B, Loehr J (2013). Small signal stability assessment of power systems with increased penetration of photovoltaic generation: A case study. IEEE Trans. Sustain. Energy.

[59] Yu, M. *et al.* Use of an inertia-less virtual synchronous machine within future power networks with high penetrations of converters. In *2016 Power Systems Computation Conference (PSCC)* 1–7 (2016).

[60] Munkhchuluun E, Meegahapola L, Vahidnia A (2020). Long-term voltage stability with large-scale solar-photovoltaic (PV) generation. Int. J. Electr. Power Energy Syst..

[61] DIgSILENT_GmbH. *DIgSILENT PowerFactory Version 2019 User Manual* (ed. DIgSILENT GmbH) (2018).

[62] Reis, C. & Barbosa, F. P. M. A comparison of voltage stability indices. In *MELECON 2006–2006 IEEE Mediterranean Electrotechnical Conference* 1007–1010 (2006).

[63] Bhonsle, J. S., Deshpande, S. B., Renge, M. M. & Harne, R. V. A new approach for determining weakest bus and voltage stability margin in a power system. In *National Power Systems Conference* 102–107 (2004).

[64] DIgSILENT_GmbH. *14 Bus System* (ed. DIgSILENT GmbH) 1–8 (2018).

[65] DIgSILENT_GmbH. *39 Bus New England System* (ed. DIgSILENT GmbH) 1–15 (2018).

[66] Jin C, Li W, Liu L, Li P, Wu X (2019). A coherency identification method of active frequency response control based on support vector clustering for bulk power system. Energies.

